# Effect of Celluclast 1.5L on the Physicochemical Characterization of Gold Kiwifruit Pectin

**DOI:** 10.3390/ijms12106407

**Published:** 2011-09-27

**Authors:** Oni Yuliarti, Lara Matia-Merino, Kelvin K. T. Goh, John A. Mawson, Charles S. Brennan

**Affiliations:** 1 Institute of Food Nutrition and Human Health, Massey University, Palmerston North 4474, New Zealand; E-Mails: oniyuliarti@yahoo.co.nz (O.Y.); l.matia-merino@massey.ac.nz (L.M.-M.); k.t.goh@massey.ac.nz (K.K.T.G.); 2 Department of Applied Science, London South Bank University, 103 Borough Road, London SE1 0AA, UK; E-Mail: mawsonj@lsbu.ac.uk; 3 Department of Wine and Molecular Biosciences, Lincoln University, Lincoln 7647, New Zealand

**Keywords:** pectin, gold kiwifruit, pectin extraction, enzyme concentration, molecular weight, galacturonic acid, viscosity

## Abstract

The effects of Celluclast 1.5L concentration on the physicochemical characterization of gold kiwifruit pectin was evaluated. Varying the enzyme concentration affected the pectin yield and pectin physicochemical properties. The viscosity of extracted pectin was largely dependent on the enzyme concentration. Celluclast 1.5L with medium concentration exhibited the highest viscosity. Varying the enzyme concentration also influenced the molecular weight distribution. High molecular weight (*M*_w_) pectin (1.65 × 10^6^ g/mol) was obtained when the medium concentration was used. Overall, the study clearly reflects the importance of taking into consideration the amount of cellulytic enzyme added in order to determine the final quality of pectin.

## 1. Introduction

Pectin is one of the main polysaccharide of the cell walls of growing plants. Pectin can be obtained in soluble form and is well known for its ability to improve food quality through its influence on texture. The composition, physicochemical, and functional properties of pectin have ensured that it is used widely as a thickener and stabilizer. Apple pomace and citrus peel are currently the two main sources of commercially available pectin, although some other sources are being studied intensively.

A major challenge in developing any new pectin product is the selection of the isolation procedures to be used and the influence of the method and the isolation conditions on the properties of the pectin. It is generally desirable to be able to preserve the physicochemical characteristics of pectin as these properties will contribute to its functionality.

The most common method for extracting pectin without further degradation is to use enzymes. Compared with acid solution, commercial enzymes are preferred despite their cost because only a little effluent is found in the filtrate and because of consumer demands for green products. However, the mechanism of enzymatic extraction is still not fully understood [1]. This research considered the use of a commercial cellulase preparation such as Celluclast 1.5L to promote the release of pectic substances.

Celluclast 1.5L contains predominantly cellulases (endo-glucanase units) and has been found to be effective in extracting pectin from plant cell walls. It breaks down the cellulosic materials in chicory cell walls and converts them to glucose. Endo-glucanase hydrolyses the cellulose chain internally and produces oligosaccharides, cellobiose and glucose [2]. Cellulose is a water-insoluble linear polymer of β-(→4) linked d-glucopyranosyl units [3]. Previous studies [4,5] have indicated that Celluclast 1.5L can be used to extract pectin from gold kiwifruit. Panouille *et al*. [1] also showed that using the enzyme together with protease (Neutrease) and Cellulyve TR400 increases the cellulose solubilization to glucose to 80% and thus improves the pectin yield. This use of Celluclast 1.5L for pectin extraction increases the pectin yield, because there is a direct interaction between cellulose and the galactan side chain of pectin.

Research information on gold kiwifruit pectin is limited and to date there is no study on the optimization of enzyme concentration to isolate gold kiwifruit pectin. Therefore, the aim of this research was to study the extraction of pectin from gold kiwifruit puree using different Celluclast 1.5L concentrations. In this study the effect of enzyme concentration on the yield of pectin and the pectin physicochemical properties extracted from gold kiwifruit are discussed.

## 2. Experimental

### 2.1. Materials

Gold kiwifruit (cv. Hort16A) used in this study was obtained from Zespri International Ltd. (Hastings, New Zealand). The fruit was main-harvested fruit (main season fruit, approximately 20 weeks after pollination, with 3.3 kilogram-force firmness) supplied from the store of a commercial processing plant in July 2007. All kiwifruits were frozen at −20 °C after harvest and were thawed (at 4 °C for approximately 18 h) prior to extraction. The whole kiwifruit puree was prepared as reported previously [4].

### 2.2. Pectin Extraction

Gold kiwifruit puree (200 g) was mixed with the Celluclast 1.5L (Novozymes, Copenhagen, Denmark) with three different enzyme concentrations, namely low (0.1 mL/kg), medium (1.05 mL/kg) and high (2.0 mL/kg). The puree samples were heated to 25 °C before adding the enzymes and incubated in a temperature-controlled water bath for 30 min with continuous stirring. The mixture was immediately cooled to approximately 3–4 °C in a bath of crushed ice for 20 min to slow further enzyme reactions and was then centrifuged (3310 g, 20 min, 4 °C) (Centra, MP4R, rotor 224, International Equipment Company, USA) to separate the insoluble fraction. The supernatant was filtered through four layers of cheese cloth to remove these particles. Tepid water (25 °C) was added to the pellet, in the ratio 1:1 (w/v), and was stirred for 30 min to recover the remaining soluble polysaccharides trapped in the pellet. The mixture was centrifuged again as before. All extracts were combined and precipitated with ethanol. Ethanol (95%) was added to the supernatant in a 5.3:1 (v/v) ratio to achieve 80% v/v ethanol. The mixture of ethanol and extract was stirred using a magnetic stirrer for about 10 min at room temperature to obtain a uniform mixture and was then kept at 4 °C [6] for 4 h to allow the polysaccharides to precipitate [7]. To separate the polysaccharide precipitate from the solvent, the mixture was centrifuged (3310 g, 10 min, 4 °C). The pellet was washed twice with 95% ethanol (1:1, w/v) and then centrifuged again as before. The pellet from the ethanol precipitation was vacuum dried (Eyela, Vacuum Oven, Voc-300 SD, Science Technique Ltd, New Zealand) at 58 ± 3 °C, 65 cm Hg, for approximately 7–10 h until a constant weight was achieved. For purification purposes, the vacuum-dried sample was dispersed in Milli-Q water (1.0% w/w) and stirred overnight (~15 h) in a 4 °C chiller. The dispersion was then centrifuged at 30,000 g for 60 min (4 °C) to separate out the insoluble fraction. The supernatant was freeze dried (FD18, Cuddon, Blenheim, New Zealand) for three days and the recovered fraction was identified as either crude pectin. The amount of crude pectin recovered was used to calculate the yield, as described by Ptitchkina, Markina and Runlyantseva [8] as the below equation. All extraction experiments in this study were performed in duplicate.

$$D=100\times \left(\frac{m_{\text{pectin}}}{m_{\text{kp}}}\right)$$

where *D* is the percentage yield of purified pectin (%), *m*_pectin_ is the mass of recovered pectin (g) and *m*_kp_ is the mass of kiwifruit puree (g) used in the extraction.

### 2.3. Analytical

The neutral sugar composition was determined by gas liquid chromatography (Hewlett Packard, 5890 and BPX-70 column), in the form of alditol acetate derivatives as described by Englyst, Quigley and Hudson [9]. Galacturonic Acid (GalA) was determined by a colorimetric method using an UV-160A spectrophotometer (Shimadzu, Douglas Scientific, Auckland), in which the difference in absorbance of a sample at 400 nm and at 450 nm was measured against a blank consisting of 2 M H_2_SO_4_. The method has been described by Scott [10].

#### 2.3.1. *M*_w_ Determination Using SEC-MALLS

Determination of the *M*_w_ of pectin was based on the SEC-MALLS technique described by Goh, Hemar and Singh [11]. The *M*_w_ and polydispersity index (*M*_w_/*M*_n_) of vacuum-dried purified pectin were determined by size exclusion chromatography (SEC) coupled to a multi-angle laser light scattering (MALLS) system (Mini Dawn, Wyatt Technology Corp., Santa Barbara, CA, USA). This system consisted of a high performance liquid chromatography (HPLC) system (GBC Scientific Equipment Ltd, Victoria, Australia), which comprised an HPLC pump (model LC 1150), an ultraviolet (UV) detector (model LC 1200), a system organizer (model LC 1440) and a differential refractive index (DRI) detector (Waters, model R401, Milford, MA, USA).

The eluant was prepared by dissolving 0.02% w/v sodium azide and 0.1 M sodium chloride (NaCl) in Milli-Q water. The solution was filtered through a 0.22 μm membrane filter (Millipore Corp., Bedford, MA, USA) followed by a 0.025 μm membrane filter (Millipore) and was degassed prior to use. The pectin samples (0.35% w/w) were dispersed in 0.1 M NaCl solution. The samples were filtered through a 0.22 μm filter prior to sample loading. Two commercial polysaccharide samples, namely dextran (Sigma, *M*_w_ ~ 6 × 10^3^ g/mol) and citrus pectin (Sigma, P9135, unknown *M*_w_) were also prepared for comparative purposes.

Separation of the molecular fraction was accomplished using a Shodex SB-805 column as a size-exclusion column connected to a guard column SB-6 (Shodex, Tokyo, Japan). The eluant was continuously gassed with helium and was pumped through the HPLC system to the SEC column at a flow rate of 0.50 mL/min at 20 psi. The eluant from the SEC column flowed through the UV detector at 280 nm, the MALLS detector and the DRI detector. The pectin sample (~100 μL) was loaded into the column through an injection port and was separated at 35 °C over an elution time of approximately 45 min. For the molar mass calculation, the specific refractive index increment (*dn*/*dc*) of the pectin fraction was determined (section below). The data were analyzed using Astra software (version 4.50, Wyatt Technology Corp., Santa Barbara, CA, USA) and the Zimm plot method to determine the *M*_w_ and *M*_w_/*M*_n_ of the pectin fraction.

#### 2.3.2. Specific Refractive Index Increment (*dn*/*dc*)

NaCl solutions at various concentrations (0.025, 0.0625, 0.125, 0.1875 and 0.25% w/w) were prepared and measured using the DRI detector to obtain a plot of DRI voltage (V) versus NaCl concentration. The measurement was conducted at 35 °C. The DRI response factor (*dV*/*dc*) was obtained from the gradient of the plot of DRI as a function of NaCl concentration. The gradient (*dV*/*dc*) was divided by the known *dn*/*dc* value of NaCl (1.74 mL/g) to obtain *dV*/*dn*. Different concentrations of purified pectin sample (1, 0.50, 0.33, 0.25 and 0.2% w/w) were prepared by dissolving the pectin (based on GalA concentration) in the same solvent as for the *M*_w_ analysis (0.1 M NaCl, 0.02% sodium azide). The slope of the pectin sample (*dV*/*dc*) was divided by the factor (*dV*/*dn*) of the calibration slope to obtain the *dn*/*dc* value of the pectin. Determination of the *dn*/*dc* value was carried out in duplicate.

#### 2.3.3. Viscosity Determination

Freeze-dried pectin samples (4% w/w dry weight) were re-dispersed in fresh Milli-Q water under continuous stirring at room temperature for 15 min, followed by stirring for 15 min at 60 °C. The samples were de-gassed in an ultrasonic water bath (for a few seconds only) to release the bubbles in the solution, and the pH was adjusted to 3.50 ± 0.01 by the slow addition of 0.5 M HCl or 0.5 M NaOH prior to the viscosity measurements. Viscosity curves were obtained using a controlled-stress rheometer (Paar Physica MCR 301; Anton-Paar, GmbH, Germany) with a cone and plate measuring system (CP 4/40) at 20 ± 0.1 °C and at shear rates between 1 and 1000 s^−1^.

### 2.4. Data Analysis

The experiment was a completely randomized design (CRD), where enzyme concentration was varied (low, medium and high) at constant extraction time (30 min) and temperature (25 °C). The data were analyzed by one-way analysis of variance (ANOVA) using the SAS program (version 9.1). Significant differences among the treatments were determined by Duncan’s multiple range test (*P* ≤ 0.05).

## 3. Results and Discussion

### 3.1. Crude Pectin Yield

Table 1 shows the yields of crude pectin extracted using three enzyme concentrations at 25 °C for 30 min. The yield is expressed as % w/w on a dry matter basis.

A significant change (*P* < 0.05) in crude pectin yield was observed when the Celluclast 1.5L concentration was varied. The yield ranged from 6.58 to 8.08%. An enzyme concentration of 1.05 mL/kg resulted in the highest yield. In contrast, the use of either a low level or a high level of enzyme showed lower pectin yields. The high enzyme concentration used could have resulted in greater pectin hydrolysis whereas the low enzyme concentration could have resulted in a low yield because insufficient enzyme was used. Compared with other plant sources, the yield of pectin from gold kiwifruit was lower than that from dried pumpkin pulp (9–14.0% w/w) and from an alcohol-insoluble residue of chicory roots (~34.6% w/w) [1,8]. These differences were attributed to the source of the raw material, the purity of the enzyme and the extraction conditions employed.

### 3.2. Sugar Compositions

Table 2 shows the total non-starch polysaccharides (NSP) and sugar compositions of the crude pectin extracted from gold kiwifruit using different Celluclast 1.5L concentrations.

As shown, GalA dominated the sugar composition of the extract. There was no significant effect of varying the Celluclast 1.5L concentration on the total-NSP and sugar compositions with the exception of rhamnose and fucose. Rhamnose was significantly influenced (*P* < 0.05) by enzyme concentration. The addition of a high level of Celluclast 1.5L (2 mL/kg) resulted in a lower rhamnose content (0.47%). This could suggest hydrolysis of rhamnogalacturonan chains induced by higher enzyme concentrations.

### 3.3. Viscosity

**Figure 1:**
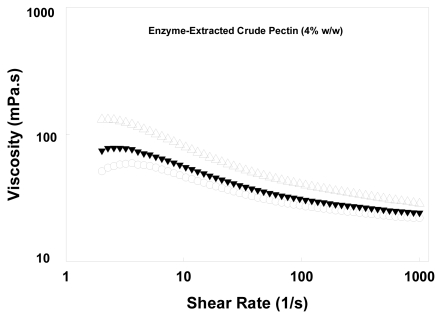
Viscosity curves of crude pectin (4% w/w dry weight in Milli-Q water, pH 3.50 ± 0.01) extracted using Celluclast 1.5L under extraction conditions of 25 °C and 30 min and at different concentrations: (○) high, (Δ) medium and (▾) low.

Figure 1 illustrates the viscosity curves of crude pectin extracted at 25 °C for 30 min using different enzyme concentrations.

The samples were prepared based on a dry weight basis of 4% w/w. The results show that pectin extracted with the medium Celluclast 1.5L concentration exhibited the highest viscosity (~46 mPa·s at 53 s^−1^) compared with the other concentrations (low and high levels). There was a slight difference in viscosity (~30 mPa·s at 53 s^−1^) for pectin samples isolated at the low and high Celluclast 1.5L concentrations. Pereyra, Schmidt and Wicker [12] reported that the *M*_w_ differences of high methoxyl pectins (HMPs) probably account for the differences in apparent viscosity.

### 3.4. The *dn/dc* and *M*_w_ of Pectin Samples

The *dn*/*dc* is the *specific refractive index increment* value which depends upon the type of polysaccharide evaluated (because different types of molecules may have different *dn*/*dc* values), the solvent and the wavelength [13]. The *dn*/*dc* values obtained for pectin extracted using Celluclast 1.5 were relatively similar (0.185–0.191 mL/g). The average *dn*/*dc* value of 0.189 ± 0.002 mL/g was used in the determination of the *M*_w_. The *dn*/*dc* value of gold kiwifruit pectin was higher than the *dn*/*dc* value of pectin from other fruits (0.146 mL/g for citrus pectin by Fishman *et al.* [14]; 0.132 mL/g for citrus pectin by Cameron *et al.* [15]). However, Corredig *et al.* [16] reported a *dn*/*dc* value for HMP from citrus of 0.183 mL/g. These authors attributed the high *dn*/*dc* value to the greater number of methoxyl groups present in the polysaccharide chains. Based on the *dn*/*dc* value determined in this study, the *M*_w_ of pectin samples was determined.

**Figure 2:**
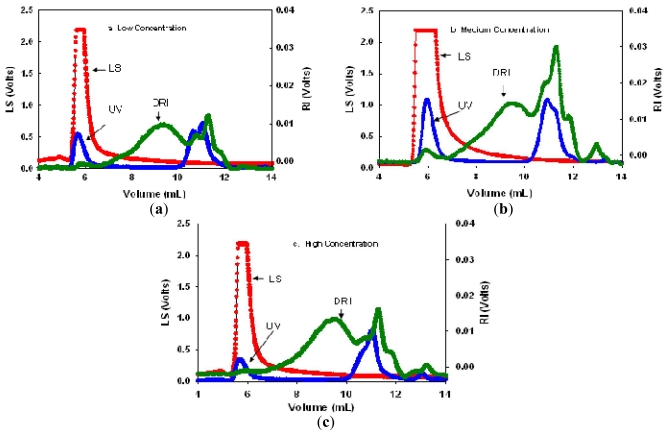
Light scattering (LS), ultraviolet (UV) and differential refractive index (DRI) signals of purified pectin extracted using different enzyme concentrations: (**a**) low; (**b**) medium; (**c**) high (based on 0.35% w/w GalA concentration, prepared in 0.1 M NaCl, 0.02% sodium azide).

Figure 2(a–c) show chromatograms of the light scattering response at 90° (LS), ultraviolet (UV) and DRI signals as a function of the elution volume (mL) from samples obtained using the different enzymatic concentrations. The DRI signal is proportional to the concentration of the polymer, the UV signal correlates to the presence of protein in the sample, whereas the LS signal depends on the size, *M*_w_ and concentration of the polymer molecules eluted through the SEC column.

The DRI profiles showed multiple peaks, indicating that the purified pectin samples consisted of more than one polymer fraction. Low DRI signals indicate a low polymer concentration. At the initial elution volume (6 mL), a small DRI signal was observed but this was accompanied by large LS and UV signals. This could have been due to the presence of a small amount of pectin aggregates (*M*_w_ *~* 10^7^–10^8^ g/mol) that eluted first.

A second wider DRI peak was observed at an elution volume of 7–12 mL. This range consisted of about four peaks with the size depending on the concentration of enzymes used in the extraction process. This signal range is believed to be due to the pectin fraction and was used to determine the *M*_w_.

A small DRI peak that eluted after 12 mL was believed to be caused by low *M*_w_ species, such as proteins, oligosaccharides and salts, which might be eluted last. The molecular fractions at the start of the elution and at the end of the elution were not considered in the *M*_w_ determination. Therefore, only the LS data at an elution volume from 7 to 12 mL were used to determine the *M*_w_s of the pectin samples. The results for the *M*_w_ and the polydispersity index are given in Table 3.

The *M*_w_ results showed that, at medium enzyme concentration, the extracted pectin had the highest *M*_w_ (~16.5 × 10^5^ g/mol). At low and high enzyme concentrations, the *M*_w_s were 3.72 × 10^5^ and 2.44 × 10^5^ g/mol, respectively. At high enzyme concentration, the pectin molecular chains were smaller, which could have been due to the side pectinolytic activities of Celluclast 1.5L. The molar mass of pectin was also lower at the low enzyme concentration than at the medium enzyme concentration. It could be possible that there was insufficient enzyme to hydrolyse the cellulose network in which the larger molar mass fraction was trapped.

Generally, the *M*_w_ of pectin is expected to be in the range 10^4^–10^5^ g/mol [15]. The *M*_w_ of commercial citrus pectin determined in this study was 1.66 × 10^5^ g/mol. This value is in close agreement with data obtained from studies on citrus pectin [17]. However, a value close to that of kiwifruit pectin, of the order of 10^6^ g/mol, has been reported for cider apple pomace pectin extracted using CDTA and Na_2_CO_3_ [18].

To explain the viscosity data described a linear regression between viscosity at 53 s^−1^ and *M*_w_ was plotted and a strong correlation (*R*^2^ = 0.99) between viscosity and molar mass was obtained. The molecular chains of pectin were influenced by the enzyme concentration, hence giving different viscosity readings.

## 4. Conclusions

The use of non starch polysaccharides in functional foods has received much interest in recent years from both a health [19] and processing [20,21] viewpoint. Pectin isolated from kiwifruit has the potential to act as a food grade non starch polysaccharide to enhance food product quality [4] The optimum concentration of Celluclast 1.5L was explored by isolating gold kiwifruit pectin at three different enzyme concentrations. The enzyme concentration had a significant effect on the yield of pectin but not on GalA concentration. Furthermore, the enzyme concentration influenced the molar mass of extracted pectin, as shown by the different viscosities. A medium concentration of the enzyme was observed to be superior compared to other concentrations as the extracted pectin exhibited the highest viscosity. Overall, the results illustrate the potential of using enzymes separately or in combination to achieve optimum extraction of pectin from whole kiwi fruit. This is of significant interest to the ingredient industry in the development of novel functional ingredients.

## Figures and Tables

**Table 1:** Effect of Celluclast 1.5L concentration on the crude pectin yield (% w/w dry matter basis) with an extraction regime of 25 °C and 30 min.

Commercial Enzyme Concentration	Crude Pectin Yield (% w/w)
Low	6.58 b
Medium	8.08 a
High	7.01 b
SEM 1	0.235
Probability	*

a b Means in a column with different superscripts differ significantly (*P* < 0.05).

1 Pooled standard error of mean.

* *P* < 0.05. Each value represents the mean of two replicates. Low (0.1 mL/kg), medium (1.05 mL/kg) and high (2.0 mL/kg).

**Table 2:** Total non-starch polysaccharides (NSP) and sugar compositions (% w/w dry weight) of crude pectin extracted from gold kiwifruit using Celluclast 1.5L at different enzyme concentrations (25 °C and 30 min).

Enzyme Concentration	Rha	Fuc	Ara	Xyl	Man	Gal	Glc	GalA	Total-NSP
Low	0.52 a	0.21 a	1.06	0.27	0.12	1.55	0.36	52.43	56.53
Medium	0.57 a	0.11 b	1.25	0.21	0.19	1.68	0.29	53.05	57.34
High	0.47 b	0.16 b	1.08	0.25	0.16	1.76	0.35	50.40	54.62
SEM 1	0.047	0.013	0.108	0.019	0.026	0.095	0.042	0.926	1.113
Probabilities	*	*	NS	NS	NS	NS	NS	NS	NS

a b Means in a column with different superscripts differ significantly (*P* < 0.05).

1 Pooled standard error of mean. NS: not significant;

* *P* < 0.05. Each value represents the mean of two replicates. Rha: rhamnose; Fuc: fucose; Ara: arabinose; Xyl: xylose; Man: mannose; Gal: galactose; Glc: glucose; GalA: galacturonic acid. Low (0.1 mL/kg), medium (1.05 mL/kg) and high (2.0 mL/kg).

**Table 3:** Average-weight molecular weight and polydispersity index of purified pectin extracted using different enzyme concentrations.

Enzyme Concentration	*M*_w_ (×10^5^ g/mol)	Polydispersity Index (*M*_w_/*M*_n_)
Low	3.72 ± 0.01	2.42 ± 0.10
Medium	16.50 ± 0.37	2.49 ± 0.06
High	2.44 ± 0.01	2.29 ± 0.17

Mean ± standard error (*n* = 2). Low (0.1 mL/kg), medium (1.05 mL/kg) and high (2.0 mL/kg).
